# *NifH* gene amplicon sequencing and metagenomic approaches are complementary in assessing diazotroph diversity

**DOI:** 10.1093/ismeco/ycaf038

**Published:** 2025-02-27

**Authors:** Shunyan Cheung, Michael Morando, Jonathan Magasin, Francisco M Cornejo-Castillo, Jonathan P Zehr, Kendra A Turk-Kubo

**Affiliations:** Institute of Marine Biology and Center of Excellence for the Oceans, National Taiwan Ocean University, 2 Beining Road, Keelung 20224, Taiwan; Ocean Sciences Department, University of California, Santa Cruz, 1156 High Street, Santa Cruz, CA 95064, United States; Ocean Sciences Department, University of California, Santa Cruz, 1156 High Street, Santa Cruz, CA 95064, United States; Department of Marine Biology and Oceanography, Institute of Marine Sciences (ICM-CSIC), Pg. Marítim Barceloneta, 37-49 08003 Barcelona, Spain; Ocean Sciences Department, University of California, Santa Cruz, 1156 High Street, Santa Cruz, CA 95064, United States; Ocean Sciences Department, University of California, Santa Cruz, 1156 High Street, Santa Cruz, CA 95064, United States

**Keywords:** diazotroph diversity, *Nifh* gene, nested PCR, amplicon sequencing, metagenomics

## Abstract

Exploring the diversity of diazotrophs is key to understanding their role in supplying fixed nitrogen that supports marine productivity. A nested PCR assay using the universal primer set nifH1-nifH4, which targets the nitrogenase (*nifH*) gene, is a widely used approach for studying marine diazotrophs by amplicon sequencing. Metagenomics, direct sequencing of DNA without PCR, has provided complementary views of the diversity of marine diazotrophs. A significant fraction of the metagenome-derived *nifH* sequences (e.g. *Planctomycete*- and *Proteobacteria*-affiliated) were reported to have nucleotide mismatches with the nifH1-nifH4 primers, leading to the suggestion that *nifH* amplicon sequencing does not detect specific diazotrophic taxa and underrepresents diazotroph diversity. Here, we report that these mismatches are mostly located in a single-base at the 5′-end of the nifH4 primer, which does not impact detection of the *nifH* genes. This is demonstrated by the presence of *nifH* genes that contain the nucleotide mismatches in a recent compilation of global ocean *nifH* amplicon datasets, with high relative abundances detected in a variety of samples. While the metagenome- and metatranscriptome-derived *nifH* genes accounted for 4.4% of the total amplicon sequence variants from the global ocean *nifH* amplicon database, the corresponding amplicon sequence variants can have high relative abundances (accounting for 47% of the reads in the database). These analyses underscore that *nifH* amplicon sequencing using the nifH1-nifH4 primers is an important tool for studying diversity of marine diazotrophs, particularly as a complement to metagenomics which can provide taxonomic and metabolic information for some dominant groups.

## Introduction

Marine diazotrophs are a diverse group of prokaryotes that fix dinitrogen (N_2_) gas to bioavailable forms of N, playing critical roles in supporting ocean productivity and biological carbon export [1]. In particular, they can contribute over half of the new production in oligotrophic waters like the North Pacific subtropical gyre [2, 3]. Historically, marine N_2_ fixation has been mostly attributed to *Trichodesmium* or diatom-associated cyanobacteria (which can be easily identified with light microscopy) [4, 5]. However, numerous marine diazotrophs have been discovered through amplicon sequencing of the *nifH* gene, which encodes the iron-containing dinitrogenase reductase subunit, and has been established as a proxy for N_2_ fixation potential. Amplification of *nifH* using the universal primer set designed by Zehr and McReynolds [6] and Zani *et al.* [7] is among the most widely used assay for obtaining *nifH* gene fragments for amplicon sequencing from the marine environment to date, in which two pairs of degenerate primers (outer primers: nifH3 and nifH4 [7]; inner primers: nifH1 and nifH2 [6]) are used in subsequent PCR reactions. This nested PCR assay targets a majority of the known *nifH* sequences from the environment [8], providing high sensitivity for detecting marine diazotrophs whose abundances are usually several orders of magnitude lower than dominant prokaryotes in the ocean [9]. This approach led to the discovery of three genetically distinct groups of unicellular cyanobacterial phylotypes, including UCYN-A (*Candidatus Atelocyanobacterium thalassa*, recently discovered as nitrogen-fixing organelles of haptophytes), UCYN-B (*Crocosphaera watsonii*) and UCYN-C [10, 11], which have been demonstrated to be widespread and quantitatively significant N_2_-fixers in the ocean [12–14]. Moreover, diverse non-cyanobacterial diazotroph (NCD) *nifH* phylotypes (i.e., putative diazotrophs) have also been detected in marine *nifH* amplicon sequence datasets, although their N_2_-fixing activity in the ocean remains unclear [15]. Recent studies discovered that two NCDs affiliated with *Alphaproteobacteria* and *Gammaproteobacteria* are active N_2_-fixers, which are widely distributed in the ocean [16, 17].

Metagenomics and metatranscriptomics, which are sequencing approaches that do not rely on amplification using specific PCR primers, have recently been used as alternative ways to obtain genomic information of marine diazotrophs [18–21]. Delmont *et al.* [19, 21] have reported dozens of metagenome-assembled genomes (MAGs) that contained *nifH* genes (and other genes for N_2_ fixation) from the seawater metagenome dataset generated by the *Tara* Oceans expedition. These analyses emphasized that some MAGs were affiliated with *Planctomycetes* (*Planctomycetota*), which was the first report of N_2_ fixation potential of this phylum in the ocean [21]. Notably, they also advised that the *nifH* genes of the *Planctomycetes*, along with some other proteobacteria, would not be detected using *nifH* amplicon sequencing approaches [19, 21], because the DNA sequences of these genes did not completely match with the universal nifH1-nifH4 primer set sequences. However, the impacts of these mismatches on the amplification of those *nifH* genes were not directly demonstrated in those studies.

Considering that *nifH* amplicon sequencing using the nifH1-nifH4 primer set has long been a fundamental approach for studying marine diazotroph populations, it is critical to clarify if this approach might be unable to detect the metagenome-derived *nifH* gene diversity as suggested [18, 19, 21]. In this study, we first conducted a thorough analysis of the nucleotide mismatches between the nifH1-nifH4 primers and the marine *nifH* sequences obtained with PCR-primer-free approaches (i.e. metagenomics, metatranscriptomics, and genome sequencing of isolates) [18, 19]. Moreover, we searched the PCR-primer-free approach derived *nifH* sequences in a global ocean *nifH* amplicon sequence variant (ASV) database recently compiled by Morando *et al.* [22], in order to compare the diversity obtained by *nifH* amplicon sequencing to that of PCR-primer-free approaches.

## Materials and methods

### Identifying nucleotide mismatches between PCR-primer-free approach derived *nifH* genes and the nifH1-nifH4 primer set

We analyzed the nucleotide mismatches between the universal nifH1-nifH4 primer set [7, 23] and the PCR-primer-free approach derived *nifH* genes. These *nifH* sequences came from the “extended *nifH* database” compiled by Delmont *et al.* [19], which contained the *nifH* gene sequences detected in the global ocean datasets of the *Tara* Oceans expedition, including the *nifH* genes of 47 MAGs (Tara MAGs) and a metatranscriptomic-based contig of Gamma A (a common marine NCD) reported from the *Tara* Oceans datasets [19, 24], and the *nifH* genes from a larger marine *nifH* database compiled by Pierella Karlusich *et al.* [14]. The *nifH* genes of the MAGs (Arctic MAGs) recently obtained by Shiozaki *et al.* [18] from the Arctic Ocean were also included in our analysis. For simplicity, the database containing all these *nifH* sequences is called the “metaGT database”. For the nine Arctic MAGs reported by Shiozaki *et al.* [18], the *nifH* gene sequences were extracted using Prokka [25]. The *nifH* gene of a MAG (Arc-Myxo) was not included in our analysis because this MAG did not contain any of the other essential genes (*nifDK* and *nifEBN*) for nitrogen fixation [18]. The *nifH* sequences in the “extended *nifH* database” and the eight Arctic MAGs were merged and aligned using the MUSCLE algorithm in MEGA 11 [26], and the short sequences that did not contain all the regions targeted by the nifH1-nifH4 primer set were removed. The nucleotide mismatches of the remaining *nifH* sequences (metaGT database, Table S1) and the nifH1-nifH4 primer set were analyzed using fuzznuc (EMBOSS version 6.6.0) [27]. To compare the frequency of the nucleotide mismatches for different taxonomic groups of diazotrophs, we analyzed the taxonomy of these *nifH* sequences at phylum level. The *nifH* genes of the MAGs and Gamma A were classified based on the taxonomy of the corresponding MAGs [17–19], while the rest of the *nifH* sequences were classified with blastX against the nr database (≥90% coverage, ≥80% amino acid similarity) [28]. Both the validly published names and the old names of the diazotroph phyla are shown in Table S2 [29].

### Searching for PCR-primer-free approach derived *nifH* genes in a global ocean *nifH* ASV database

In order to test if the nifH1-nifH4 primer set can amplify the *nifH* genes derived from the PCR-primer-free approach that mismatched with the universal *nifH* primers, sequences of the nucleotide mismatch-containing *nifH* genes were used to search against a global ocean *nifH* ASV database complied by Morando *et al.* using BLASTN [22, 30]. This database contains the *nifH* ASVs generated by reanalyzing published *nifH* amplicon sequence datasets compiled from different regions of the ocean [22]. Distribution and relative abundances of the ASVs that showed ≥99% and 100% similarities with the nucleotide mismatch-containing *nifH* gene sequences were displayed on a world map using ggplot2 in R [31, 32].

We also evaluated the contributions (in terms of proportions of all reads and ASV numbers) of the *nifH* genes (54 sequences in total) of the MAGs (Tara MAGs) and the contig of Gamma A (Tara Gamma A) from *Tara* Oceans datasets and the MAGs (Arctic MAGs) from Arctic Ocean [18, 19, 24] in surface dataset (sampling depth ≤150 m) of the global ocean *nifH* ASV database [22]. The ASVs in the database were used to search against these *nifH* genes using BLASTN [30]. The ASVs showing ≥95% DNA similarity with these *nifH* genes were classified as Tara MAGs, Tara Gamma A, or Arctic MAGs. This criterion was chosen to reflect similar thresholds implemented to recruit metagenomic/metatranscriptomic reads for these *nifH* genes in previous studies [18, 24].

## Results and discussion

### Nucleotide mismatches in the nifH4 primer are unlikely to prevent amplification of *nifH* genes

**Figure 1:**
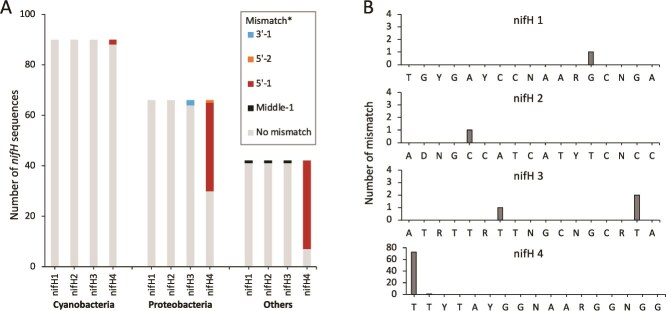
Nucleotide mismatches between the nifH1-nifH4 primers and the PCR-primer-free approach derived *nifH* genes in the metaGT database. (A) Number of *nifH* sequences affiliated to different diazotroph groups (cyanobacteria, proteobacteria, and others) that show mismatches with the nifH primers. The group “others” contains *Planctomycetes* (*Planctomycetota*), *Desulfobacterota*, *Bacteroidetes* (*Bacteroidota*), *Firmicutes* (*Bacillota*), *Verrucomicrobia* (*Verrucomicrobiota*), etc.. The number of *nifH* sequences for each diazotroph phylum is shown in Table S2. (B) Number of nucleotide mismatches that occur along each primer. *Middle-1 = 1 mismatch at the middle of primer; 5′-1 = 1 mismatch at 5′ end of primer; 5′-2 = 2 mismatches at 5′ end of primer; 3′-1 = 1 mismatch at 3′ end of primer.

By comparing the sequences of the nifH1-nifH4 primers and the *nifH* sequences in the metaGT database [18, 19, 24], we found that 37% of the *nifH* sequences had mismatches with at least one of the nifH1-nifH4 primers, and that the nucleotide mismatches were more frequently observed in NCDs (e.g. *Proteobacteria*, *Planctomycetes* (*Planctomycetota*), *Bacteroidetes* (*Bacteroidota*), *Desulfobacterota*, etc.) than in cyanobacterial diazotrophs (Fig. 1), which agreed with the previous studies [18, 19]. Importantly, most mismatches (72/78) were at a single base located at the 5’end of the nifH4 primer (Fig. 1, Table S2). Notably, two of the phylotypes identified to have such mismatches located at the 5’end of the nifH4 primer sequence in the metaGT database were the marine proteobacterial diazotrophs Gamma A (NifH_Gamma-24774A11_MATOU) and Gamma 4 (NifH_TARA_PON_109_MAG_00010), which are commonly reported in amplicon sequencing studies. Gamma A and Gamma 4 are *Alphaproteobacteria* and *Gammaproteobacteria*, respectively [17, 21, 33]. Both of these diazotrophs were first discovered with *nifH* amplicon sequencing using the nifH1-nifH4 primer set [34, 35]. Moreover, the *nifH* gene of Gamma A has been found to be preferentially amplified by the nifH1-nifH4 primer set [36]. It has been well established that primer binding is most critical in the 3′ region, given the nature of replication using DNA polymerases (5′ to 3′) and direct demonstrations that primer mismatches at 5′ end (and the nearby region) do not significantly affect the amplification efficiency of PCR reactions targeting *16S rRNA* genes [37]. Therefore, it is expected that mismatches at the 5′ end of the nifH4 primer also do not prevent the amplification of *nifH* genes with the nifH1-nifH4 primer set.

Nucleotide mismatches between the *nifH* sequences in the MetaGT database and the nifH1-nifH4 primer set at other locations in the primer sequence were also found. The 5 (out of 6) *nifH* sequences with additional mismatches were primarily single-base as well, occurring at the second base at the 3′ end of the primer nifH3 (2/78) and at the middle of the primers nifH1 (1/78), nifH2 (1/78), and nifH3 (1/78). Finally, one 2-base nucleotide mismatch was found at the first and third bases at the 5′ end of the primer nifH4 (Fig. 1, Table S2). The impact of these nucleotide mismatches, especially those occurred at the 3′ end of primer, should be further verified with PCR experiments [37].

### *NifH* genes with primer nucleotide mismatches are detected in *nifH* amplicon-based studies

**Figure 2:**
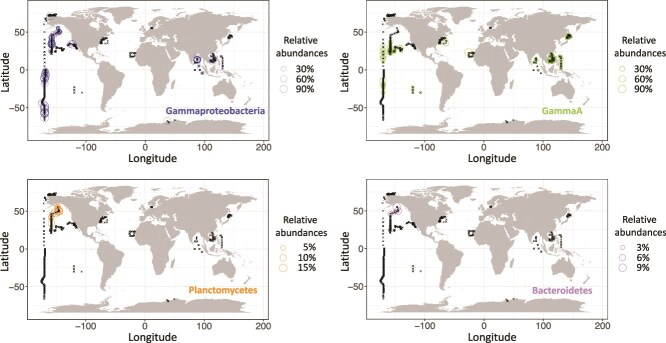
Distribution and relative abundances of the ASVs that show 99%–100% DNA similarity with the “nucleotide-mismatched” PCR-primer-free approach derived *nifH* genes (contain nucleotide mismatches with nifH primers) in the global ocean. These ASVs were grouped based on their taxonomy (i.e. *Gammaproteobacteria*, Gamma A, *Planctomycetes* (*Planctomycetota*), and *Bacteroidetes* (*Bacteroidota*)). Relative abundances of the ASVs are displayed with the sizes of data points. The sampling location for each *nifH* amplicon dataset is indicated with a cross.

In the global ocean *nifH* ASV database compiled by Morando *et al.* [22], there were a total of 79 out of 7909 ASVs with ≥99% DNA similarity to 21 of the 73 *nifH* phylotypes that contain nucleotide mismatches with nifH primers in the metaGT database. These were comprised of sequences from *Gammaproteobacteria* (51/79), *Alphaproteobacteria* (i.e. Gamma A, 21/79), *Desulfobacterota* (3/79), *Bacteroidetes* (2/79), *Betaproteobacteria* (1/79), and *Planctomycetes* (1/79) (Table S3), which all have one base-mismatches with the 5’end of the nifH4 primer. Except for one *Desulfobacterota*- and few *Gammaproteobacteria*-affiliated ASVs only detected in one sample, these ASVs were detected repeatedly, often with high relative abundances in the global ocean *nifH* ASV database (Fig. 2, Table S3) [22]. Some *Gammaproteobacteria*- and *Planctomycetes*-affiliated ASVs reached relative abundances of 100% and 16%, respectively (Fig. 2, Table S3). We also found that 22 out of these 79 ASVs are identical (100% DNA similarity) to the *nifH* genes that contain nucleotide mismatches with nifH primers, which are affiliated to *Gammaproteobacteria*, Gamma A, *Desulfobacterota*, *Betaproteobacteria*, and *Planctomycetes* (Table S3). These ASVs can reach high relative abundances in the *nifH* ASV database (Fig. S1). Collectively, these results reinforced that the most predominant type of nucleotide mismatch (single base at 5′ end of the nifH4 primer) between the *nifH* gene sequences and the nifH1-nifH4 primers do not prevent the amplification of these sequence types using the nifH1-nifH4 primer set from the environment.

It is important to note that *nifH* sequences with non-5′-end primer nucleotide mismatches (e.g. single base at 3’end or middle) were not detected in the *nifH* ASV database. These kinds of mismatches can impact the amplification significantly [37]. However, very few of the *nifH* genes in the metaGT database have these kinds of mismatches (Fig. 1). Considering that the distributions of diazotrophs are known to be highly patchy in the ocean [38, 39] and the sampling sites of the metagenomic studies had little overlap with sampling sites in the *nifH* ASV database [18, 19, 22], it is possible that these undetected *nifH* sequences (i.e. the nucleotide mismatch-containing *nifH* sequences from the metaGT database that were not detected in the *nifH* ASV database) were simply not present or very rare in the samples used for the *nifH* ASV database. Moreover, distributions of the metagenome assembled *nifH* genes were also highly heterogenous in the *Tara* Oceans datasets [19]. All in all, it remains unclear if the nifH1-nifH4 primers can amplify these non-5′-end primer nucleotide mismatch-containing *nifH* genes. To evaluate the amplification efficiency of the nucleotide mismatch-containing *nifH* genes with nifH1-nifH4 primers, the DNA samples in which the nucleotide mismatch-containing *nifH* genes were detected with metagenomics [19] would need to be further analyzed with *nifH* amplicon sequencing.

### Amplicon sequencing as a valuable approach to explore diazotroph diversity

**Figure 3:**
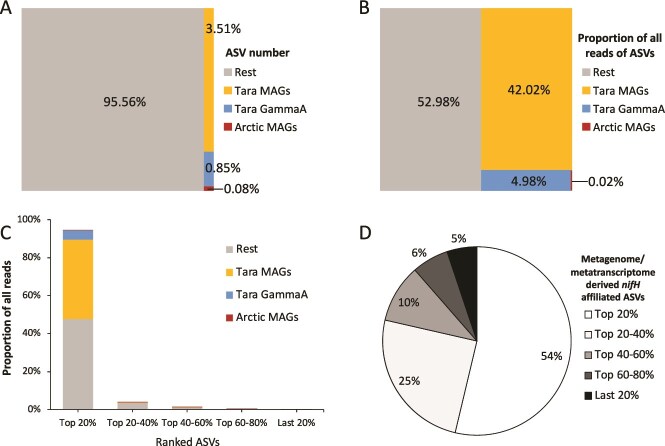
The metagenome/metatranscriptome-derived *nifH* affiliated ASVs in the global ocean *nifH* ASV database (sampling depth ≤ 150 m). The ASVs in the *nifH* amplicon database were grouped into four categories based on DNA similarity (≥95%) to the metagenome/metatranscriptome-derived sequences: the ASVs affiliated to the MAGs (Tara MAGs [19]) and a metatranscriptome-derived contig of Gamma A from the *Tara* oceans dataset (Tara Gamma A [24]); the ASVs affiliated to the MAGs from the Arctic Ocean (Arctic MAGs [18]) and the rest ASVs (rest). (A) Percentages of ASVs contributed by different ASV groups in the *nifH* ASV database. (B) Proportion of all reads of different ASV groups in *nifH* ASV database. (C) Proportion of all reads of different ASV groups among top 20%, top 20–40%, top 40–60%, top 60–80%, and last 20% ASVs in the *nifH* ASV database. (D) Percentages of the metagenome/metatranscriptome-derived *nifH* affiliated ASVs that belong to the top 20%, top 20–40%, top 40–60%, top 60–80%, and last 20% ASVs.

Considering that the Tara MAGs were reported to contribute the vast majority (92%) of the *nifH* reads detected in surface and deep chlorophyll maximum samples in the *Tara* Oceans datasets [19], for comparison, we determined the contribution of the *nifH* genes (54 sequences in total) of the Tara MAGs, Gamma A contig from the *Tara* Oceans metatranscriptomic dataset [24] (the MAG was reconstructed in a recent study [17]) and Arctic MAGs to the ASV numbers and reads in the surface layer samples (sampling depth ≤150 m) in global ocean *nifH* ASV database. As a result, 29 of these *nifH* genes were detected in the *nifH* ASV database, accounting for 4.4% of the total ASVs (350 out of 7887 ASVs) (Fig. 3A). The Tara MAGs, Gamma A, and Arctic MAGs contributed 3.5%, 0.85%, and 0.08% of the total ASVs, respectively. These results may be influenced by different sampling locations and efforts in the studies based on different approaches [18, 19, 22]. Nevertheless, it is obvious that *nifH* amplicon sequencing can detect highly diverse marine *nifH* phylotypes, for which the genomes (or MAGs) remain mostly unknown.

We also compared the detection of different categories of *nifH* genes (cyanobacterial diazotrophs vs NCDs; “matched” vs “mismatched” Tara MAGs of NCDs) with metagenomics and *nifH* amplicon sequencing (Figs S2 and S3). We detected similar proportions of cyanobacterial diazotrophs and NCDs in the *Tara* Oceans metagenomic dataset (43%: 57%) and the *nifH* ASV database (39%: 60%) (Fig. S2). For the NCD MAGs (including the contig of Gamma A) reconstructed from the *Tara* Oceans datasets, we also detected comparable proportions of the *nifH* genes that match and mismatch with the nifH primers in the *Tara* Oceans metagenomic dataset (12%: 88%) and the *nifH* ASV database (1%: 99%) (Fig. S3). The amplification efficiency of different categories of *nifH* genes should be further verified by analyzing the same environmental DNA samples with both *nifH* amplicon sequencing and metagenomics.

When considering the merits of PCR-based vs. PCR-primer-free approaches for studying diazotroph diversity, it is important to consider that marine diazotrophs are usually rare species compared to other dominant non-diazotrophic microbes in the ocean [9], and the contribution of their genomic DNA to the total DNA pool is minor compared to non-diazotrophic microbes. Thus, deep sequencing is needed to recover their genomes using metagenomic-based approaches. This problem may also be exacerbated for particle-attached NCDs whose reads can be masked by that of eukaryotic cells (with more genomic DNA) during metagenomic sequencing. For example, the *nifH* reads from Gamma A (mostly detected in large planktonic size-fraction DNA samples >5 μm, recently found to be the symbionts of diatoms [17]) were less abundant than that of Gamma 4 in the *Tara* Oceans datasets [19, 24], while their absolute abundances may often be similar based on qPCR analysis [33]. Moreover, in the *Tara* Oceans datasets, an almost complete MAG of Gamma 4 (5 532 770 bases) was obtained, but only a short contig of Gamma A (4737 bases) was obtained. Marine particles have been proposed to be important hotspots for some NCDs [40], while the diversity and genomes of particle-attached diazotrophs are difficult to access using metagenomic sequencing, as mentioned above. In contrast, our results indicate that *nifH* amplicon sequencing is a sensitive approach for assessing the diversity of diazotrophs in environmental samples (Fig. 3B). Moreover, high throughput amplicon (primer-based) sequencing is also cost-effective relative to metagenomic/metatranscriptomic sequencing, and a user-friendly pipeline exists for the bioinformatic processing of *nifH* amplicon sequencing [22]. On the other hand, the metagenome/metatranscriptome-sourced *nifH* affiliated ASVs mostly showed high relative abundance (Fig. 3C and D) that collectively contributed 47% of the reads in the global ocean *nifH* ASV database (Fig. 3B, Table S4), suggests that metagenomic sequencing can generally capture the genetic diversity of abundant diazotrophs.

Metagenomics and metatranscriptomics are among the many approaches for studying marine diazotrophs. In addition to providing critical information of the metabolic potentials of diazotrophs, these MAGs also provide taxonomic information for the unidentified *nifH* genes discovered with amplicon sequencing, especially the cluster III *nifH* phylotypes that branch deeply (e.g. *nifH* genes of *Planctomycetes* and *Verrucomicrobiota*) [15]. In conclusion, our analysis indicates that *nifH* amplicon sequencing using the nifH1-nifH4 primer set remains an important approach for studying the diversity of marine diazotrophs in terms of coverage and sensitivity, while metagenomic sequencing provides metabolic and taxonomic information on the abundant diazotrophs.

## Supplementary Material

Supplementary_materials_ycaf038

Table_S1_ycaf038

Table_S2_ycaf038

Table_S3_ycaf038

Table_S4_ycaf038

## Data Availability

The global ocean *nifH* ASV database (version 1) analyzed in this study is available in Figshare (https://doi.org/10.6084/m9.figshare.23795943.v1).
