# Supplementary material for: NifH gene amplicon sequencing and metagenomic approaches are complementary in assessing diazotroph diversity
Source: ISME Commun. 2025 Feb 27;5(1):ycaf038. doi: 10.1093/ismeco/ycaf038 (PMC11964895; doi:10.1093/ismeco/ycaf038)
Supplement: Supplementary_materials_ycaf038 [file supplementary_materials_ycaf038.pdf]

Supplementary materials for

NifH gene amplicon sequencing and metagenomic approaches are complementary in assessing diazotroph diversity

Shunyan Cheung[1+*], Michael Morando[2+], Jonathan Magasin[2], Francisco M. Cornejo-Castillo[3], Jonathan P. Zehr[2], Kendra A. Turk-Kubo[2*]

[1]Institute of Marine Biology and Center of Excellence for the Oceans, National Taiwan Ocean University, Keelung 20224, Taiwan

[2]Ocean Sciences Department, University of California, Santa Cruz, 1156 High Street, Santa Cruz, CA 95064, United States

[3]Department of Marine Biology and Oceanography, Institute of Marine Sciences (ICM-CSIC), Pg. Marítim Barceloneta, 37-49 08003 Barcelona, Spain

[+] Authors contributed equally.

[*] Corresponding authors: Kendra A. Turk-Kubo (kturk@ucsc.edu), Ocean Sciences Department, University of California, Santa Cruz, 1156 High Street, Santa Cruz, California 95064, United States; and Shunyan Cheung (sycheungisaac@mail.ntou.edu.tw), Institute of Marine Biology, National Taiwan Ocean University, Keelung 20224, Taiwan.

This supplementary file includes:

Supplementary text

Fig. S1-S3

Table S1-S4

References

Supplementary text

Comparing the proportions of different diazotroph categories detected with metagenomics and nifH amplicon sequencing

We compared the proportions of cyanobacteria diazotrophs and NCDs detected in the *Tara* Oceans metagenomic dataset [1] and the global ocean *nifH* ASV database [2]. The taxonomic information (i.e., cyanobacteria or NCDs) and the read abundances of the *nifH* ASVs came from the global ocean *nifH* ASV database, in which the ASVs were classified with *blastx* (smallest E-value among alignments with ≥50 % amino acid identity and ≥90 % query sequence coverage) against a database containing the *nifH* amino acid sequences from 879 diazotroph genomes ("genome879", https://www.jzehrlab.com/file-share/a9f22603-4bbb-4147-9293-c0b19d6fcbd9) [2]. For comparison, the *nifH* genes that had been detected in the *Tara* Oceans metagenomic dataset [1] were also classified with the approach as mentioned above. The abundances of the metagenomic reads that are affiliated to these *nifH* genes are available in paper published by Delmont et al. [1].

We also compared the proportions of "*Tara* NCD MAGs" *nifH* genes that match and mismatch with the *nifH* primers (Table S2) in the *Tara* Oceans metagenomic dataset [1] and the global ocean *nifH* ASV database [2]. "*Tara* NCD MAGs" means the NCD-affiliated MAGs and the metatranscriptome-derived contig of Gamma A reconstructed from the *Tara* Oceans dataset. Abundances of the reads that are affiliated to the *nifH* genes of these *Tara* NCD MAGs in the *Tara* Oceans metagenomic dataset and the global ocean *nifH* ASV database are available in the paper published by Delmont et al. [1] and Table S4 of this paper, respectively.

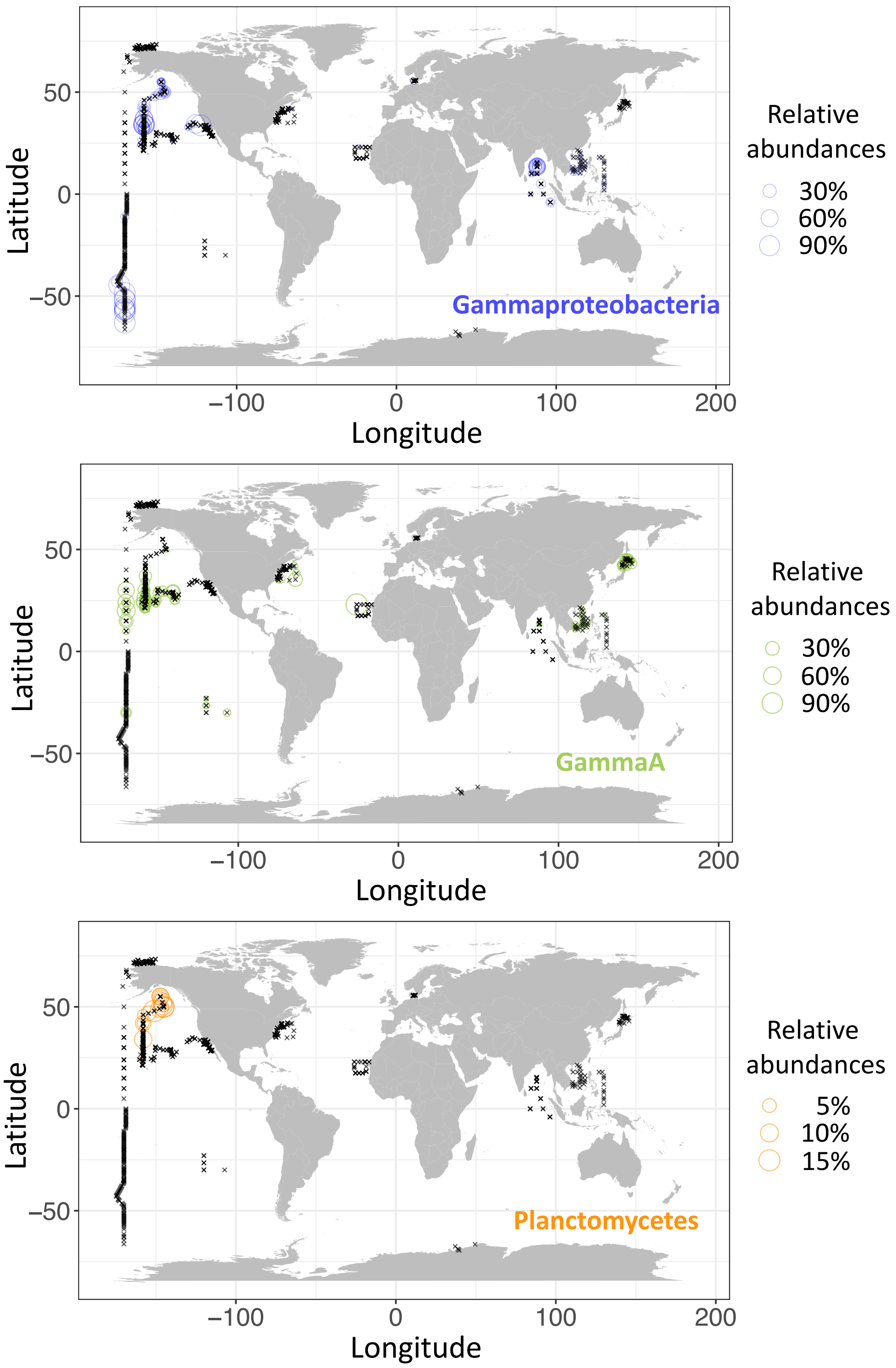

Fig. S1. Distribution and relative abundances of the amplicon sequence variants (ASVs) that show 100% DNA similarity with the "nucleotide-mismatched" PCR-primer-free approach derived *nifH* genes (contain nucleotide mismatches with nifH primers) in the global ocean. These ASVs were grouped based on their taxonomy (i.e., Gammaproteobacteria, Gamma A, Planctomycetes). Relative abundances of the ASVs are displayed with the sizes of data points. The sampling location for each *nifH* amplicon dataset is indicated with a cross.

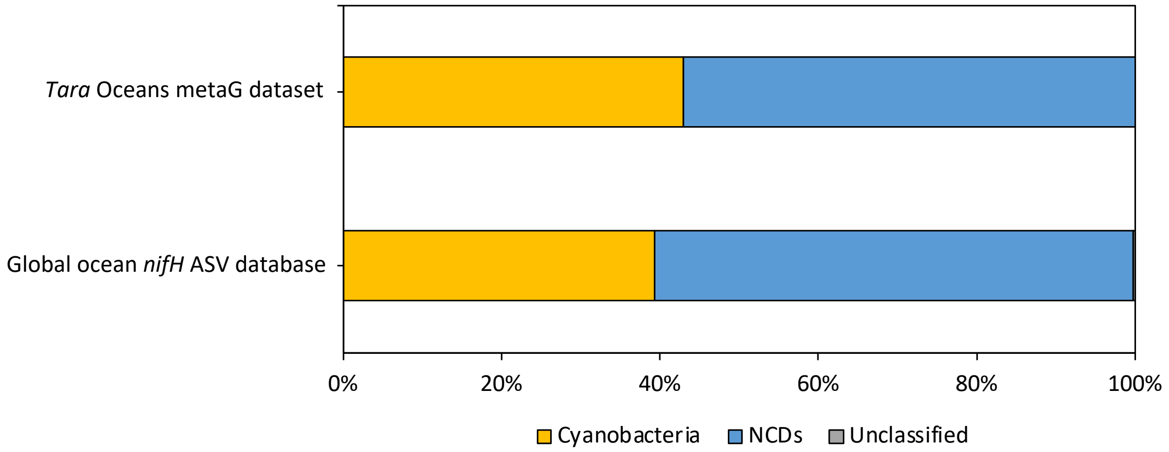

Fig. S2. Proportions of the reads mapping to cyanobacterial diazotrophs and non-cyanobacterial diazotrophs (NCDs) in the *Tara* Oceans metagenomic (metaG) dataset and the surface dataset (sampling depth ≤ 150m, Table S4) of the global ocean *nifH* ASV database.

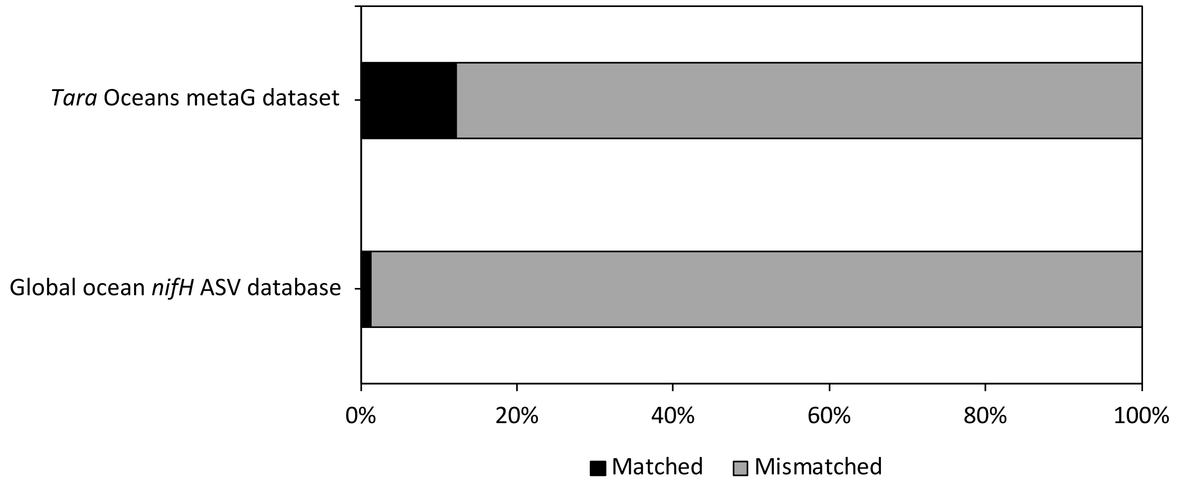

Fig. S3. Proportions of the reads mapping to the *nifH* genes of "*Tara* NCD MAGs" (i.e., the NCD-affiliated MAGs and the metatranscriptome-derived contig of Gamma A reconstructed from the *Tara* Oceans dataset) that matched and mismatched with the nifH1-nifH4 primers in the *Tara* Oceans metagenomic (metaG) dataset and the global ocean *nifH* ASV database.

Table S1. *NifH* sequences in the metaGT database. The regions in these sequences that are targeted by nifH1, nifH2, nifH3, and nifH4 primers are highlighted with red, orange, blue and brown color, respectively.

Table S2. Nucleotide mismatches between nifH1-4 primers and the *nifH* sequences in the metaGT database. The nucleotide mismatches are highlighted with red color. The rows for the *nifH* genes of the MAGs reconstructed by Delmont et al. (2022) and Shiozaki et al. (2023) were highlighted with yellow and blue color, respectively. Both the validly published names (in brackets) and the old names of the diazotroph phyla are shown in the column of "Taxonomic information (phylum)". 0 = no mismatch; M1 = 1 mismatch at the middle of primer; 5'-1= 1 mismatch at 5' end of primer; 5'-2 = 2 mismatches at 5' end of primer; 3'-1= 1 mismatch at 3' end of primer. *The sequences of reverse primers nifH2 and nifH3 were reverse and complement.

Table S3. Relative abundances of the ASVs showing ≥99% DNA similarity with the "nucleotide-mismatched" *nifH* genes. The 22 ASVs that are identical to "nucleotide-mismatched" *nifH* genes (100% DNA similarity) are labeled with asterisks. The ASVs are named with AUID (e.g., AUID.19), and the corresponding "nucleotide-mismatched" *nifH* genes (e.g., Arc-Bactero) and the taxonomic groups (i.e., Bacteroidetes, Chlorobiota, Betaproteobacteria, Desulfobacterota, Alphaproteobacteria, Planctomycetes and Gammaproteobacteria) are displayed underneath the AUIDs.

Table S4. Count table of ASVs in the surface dataset (sampling depth ≤ 150m) of the global ocean *nifH* ASV database. The ASVs are named with AUIDs (e.g., AUID.112). The taxonomic information, matched (DNA similarity ≥ 95%) metagenome/metatranscriptome assembled *nifH* genes (e.g., Arc-Alpha) and the corresponding groups (i.e., Tara MAGs, Tara GammaA and Arctic MAGs) are also displayed. The taxonomic information of the AUIDs came from the

global ocean *nifH* ASV database [2]. *The metagenome/metatranscriptome assembled *nifH* genes that mismatch with the nifH primers are labeled with asterisks.

References

1. Delmont TO, Pierella Karlusich JJ, Veseli I, Fuessel J, Eren AM, Foster RA, *et al.* Heterotrophic bacterial diazotrophs are more abundant than their cyanobacterial counterparts in metagenomes covering most of the sunlit ocean. *ISME J*. 2022; **16**:927-36.
2. Morando M, Magasin JD, Cheung S, Mills MM, Zehr JP, Turk-Kubo KA. Global biogeography of N_2-fixing microbes: *NifH* amplicon database and analytics workflow. *Earth Syst Sci Data*. 2025;**17(2)**: 393-422.
